# Organization, Function, and Therapeutic Targeting of the Morbillivirus RNA-Dependent RNA Polymerase Complex

**DOI:** 10.3390/v8090251

**Published:** 2016-09-10

**Authors:** Julien Sourimant, Richard K. Plemper

**Affiliations:** Institute for Biomedical Sciences, Georgia State University, 100 Piedmont Av, Atlanta, GA 30303, USA; jsourimant@gsu.edu

**Keywords:** measles virus, morbillivirus, viral polymerase, antiviral drug, polymerase inhibitor

## Abstract

The morbillivirus genus comprises major human and animal pathogens, including the highly contagious measles virus. Morbilliviruses feature single stranded negative sense RNA genomes that are wrapped by a plasma membrane-derived lipid envelope. Genomes are encapsidated by the viral nucleocapsid protein forming ribonucleoprotein complexes, and only the encapsidated RNA is transcribed and replicated by the viral RNA-dependent RNA polymerase (RdRp). In this review, we discuss recent breakthroughs towards the structural and functional understanding of the morbillivirus polymerase complex. Considering the clinical burden imposed by members of the morbillivirus genus, the development of novel antiviral therapeutics is urgently needed. The viral polymerase complex presents unique structural and enzymatic properties that can serve as attractive candidates for druggable targets. We evaluate distinct strategies for therapeutic intervention and examine how high-resolution insight into the organization of the polymerase complex may pave the path towards the structure-based design and optimization of next-generation RdRp inhibitors.

## 1. Introduction

**Figure 1:**
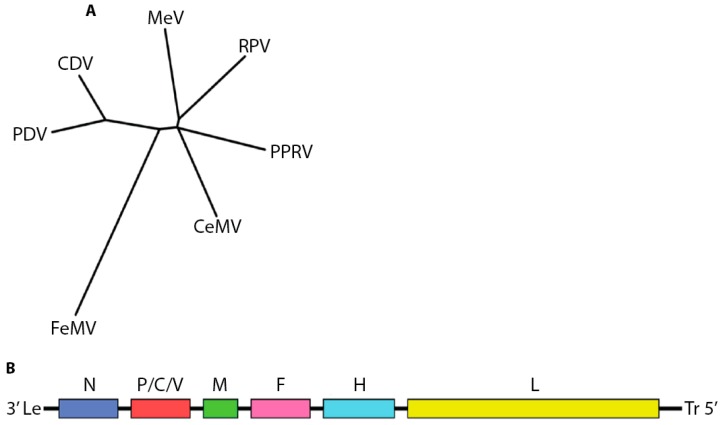
Overview of the morbillivirus genus. (**A**) Phylogenetic analysis of amino acid sequences of nucleocapsid genes of different morbillivirus reference strains. The unrooted tree was constructed using the neighbor-joining method. Branch lengths are relative and proportional to the number of substitutions. The unit of branch length is the number of substitutions (excluding gaps) at each given nucleotide position. Alignments were made with ClustalW2 [2,3,4] and trees rendered with Drawtree from the PHYLIP package 3.67 [5,6]. Genbank accession IDs: FeMV JQ411014; MeV NC_001498; PDV P35944; PPRV NC_006383; CeMV NC_005283; CDV NC_001921; RPV YP_087120.2); (**B**) Cartoon of morbillivirus genome organization.

The paramyxovirus family encompasses a broad and diverse range of human and animal pathogens. In addition to the morbillivirus genus, the family includes the aquaparamyxo-, avula-, ferla-, henipa-, respiro-, and rubulaviruses. All spread through the respiratory route, but the morbilliviruses are characterized by extremely high infection rates. The genus includes the exclusively human-tropic measles virus (MeV) as well as pathogens infecting terrestrial carnivores (canine distemper virus, CDV), felines (feline morbillivirus, FeMV), livestock (Peste-des-petits-ruminants virus, PPRV, and Rinderpest virus, RPV), or marine mammals (cetacean morbillivirus, CeMV, and phocine distemper virus, PDV) [1] (Figure 1A).

MeV is responsible for major pediatric morbidity and mortality, and is considered to belong to the most infectious viral pathogens identified to date [7,8,9]. A safe and effective live-attenuated vaccine is available, but the high transmissibility of the virus requires approximately 95% herd immunity to suppress sporadic outbreaks [10], a level that has not yet been reached in many geographic areas [11]. Several factors are hindering the required 100% vaccination coverage that is necessary to compensate for a low rate of primary vaccination failure and induce this degree of herd immunity. Predominantly, these include limited access to the vaccine in parts of the developing world and a growing refusal of vaccination due to parental concerns and/or religious beliefs [12,13]. As a consequence, MeV remains endemic in large geographical areas and is responsible for substantial morbidity worldwide [11].

Whereas increasing vaccination coverage will be vital towards stopping endemic transmission and suppressing measles, complementing vaccination with an effective antiviral drug for post-exposure prophylaxis may allow for the rapid suppression of local MeV outbreaks in areas with overall good vaccination coverage, such as North America [14], and cut short a potential prolonged endgame for global MeV eradication that tests political will and public resolve [15,16].

The paramyxovirus RNA dependent RNA polymerase (RdRp) complex is increasingly recognized as an attractive target for therapeutic intervention [17,18]. In addition to evaluating recent progress towards the fundamental understanding of the spatial organization and function of the morbillivirus polymerase, we will assess in this review candidate druggable target sites and discuss the characterization and developmental status of selected morbillivirus RdRp inhibitors.

## 2. The Morbillivirus RNA Dependent RNA Polymerase (RdRp) Complex

Morbilliviruses possess 15 to 16 kilobase RNA genomes of negative polarity that contain six open reading frames, leading to the expression of eight proteins: the structural nucleocapsid- (N), phospho- (P), matrix (M), fusion (F), hemagglutinin (H) and large (L) proteins, as well as the non-structural V and C proteins (Figure 1B).

The coding regions of the RNA genome are flanked by 3′ leader and 5′ trailer sequences that are necessary for the initiation of transcription and replication [19,20]. Furthermore, the six open reading frames are separated by intergenic junctions containing signal elements for the initiation and termination of transcription [21]. Coding and non-coding regions are implicated in virulence [22]. Additionally, like many other members of the paramyxovirus family, morbilliviruses strictly adhere to a “rule of six”, meaning that the total genome length represents a multiple of six [21,23,24].

Upon viral entry into a cell, the RdRp complex is responsible for all polymerase activity involving the viral genome, thus functioning as a transcriptase synthesizing viral mRNAs and a replicase generating positive polarity antigenomes and progeny genomes [25]. In addition to host cell cofactors, the RdRp complex is comprised of the viral L protein, which mediates all enzymatic activities, and the P protein as its mandatory cofactor. The RNA genomes and antigenomes are encapsidated by the viral N protein during synthesis, and only these N:RNA ribonucleoprotein complexes are recognized as templates for the polymerase complex [26]. Cotranslational encapsidation of nascent genomes and antigenomes mandates that a sufficiently large pool of N proteins must accumulate in an infected cell before productive replication can occur.

During the first hours after infection, transcriptase activity of the RdRp complex leads to the linear accumulation of viral mRNAs. In this primary transcription phase, the L-P transcriptase complex initiates transcription from the 3′ leader sequence to produce predominantly monocistronic mRNAs. At each intergenic junction, a gene end (GE) sequence triggers the transcriptase to add a non-templated poly-A tail to the nascent mRNA, then stop transcription and transition to the next downstream gene start (GS) sequence, which initiates transcription to resume. Accidental detachment of the advancing transcriptase complex from the N:RNA template at GE sequences can occur, resulting in premature termination, causing a transcription gradient of viral mRNAs in which mRNAs located closer to the 3′ entry point of transcription are predominantly transcribed [27].

Towards the end of the primary transcription phase, the accumulation of newly synthesized L and P proteins leads to an increase in the number of functional RdRp complexes and, consequently, an exponential increase in the rate of viral transcription. Driven by the accumulation of newly synthesized N proteins, a switch of RdRp from transcriptase to replicase ensues that initiates the synthesis of encapsidated antigenomes and, subsequently, progeny genomes. The bulk of viral proteins are produced at a later stage of infection, when secondary transcription off of newly synthesized genomes triggers an exponential increase in the concentration of viral mRNAs.

Efficient viral transcription and replication typically requires homotypic N, P and L proteins, although in some cases heterotypic combinations between viruses of the same genus are functional [28,29]. Phylogenetic analyses across different morbilliviruses have revealed a closer evolutionary distance between subgroups of the genus such as CDV with PDV, and RPV with MeV that is consistent with the heterotypic activity profiles [30,31]. However, activity of chimeric RdRp complexes is restricted to minireplicon systems, in which plasmid-encoded viral N, P, and L proteins are co-expressed with a reporter RNA, consisting of a reporter gene flanked by the viral 3′ and 5′ leader and trailer segments [32,33]. By contrast, it has not been possible to rescue a chimeric virus with a heterogeneous combination of N, P, and L, indicating that assembly into functional complexes and RdRp interaction with the N:RNA template involves specific protein-protein interactions.

### 2.1. Nucleocapsid (N) Protein

Encapsidation of nascent genome and antigenomic viral RNA by the N protein reduces triggering of the host cell innate immune response. In vitro expression of N proteins in bacterial, mammalian or insect cell systems revealed its ability to self-assemble into flexible helical ring and/or herringbone-like structures that, in the case of morbillivirus N, have been identified by negative stain electron microscopy for RPV, PPRV and MeV [34,35].

**Figure 2:**
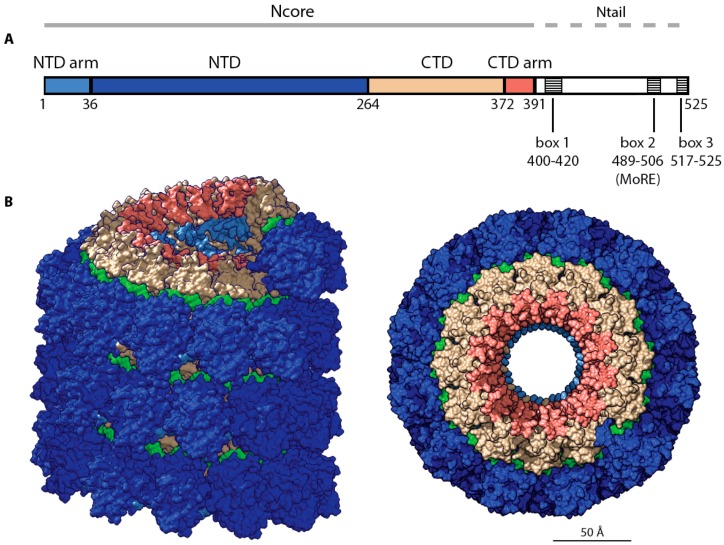

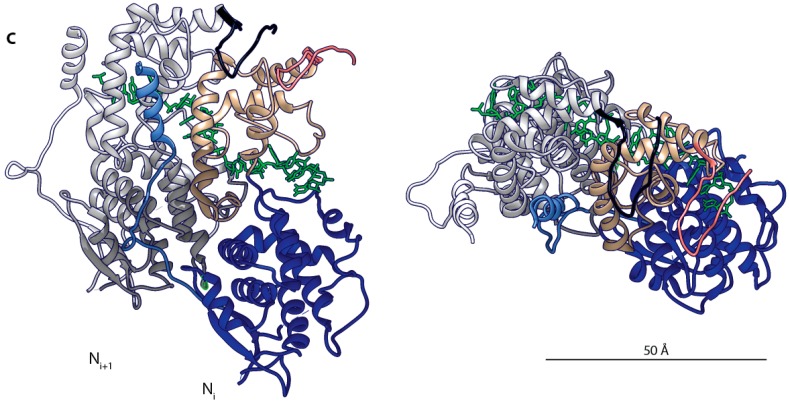
Measles virus (MeV) nucleocapsid architecture. (**A**) The nucleocapsid (N) protein is composed of an organized domain (Ncore) comprising the NTD arm (light blue, residues 1–36), NTD (blue, 37–265), CTD (tan, 266–371) CTD arm (salmon, 372–391), and a mainly disordered domain (Ntail) comprising three short conserved boxes with the α-MoRE (yellow, 488–499) located in box 2; (**B**) Surface representation of a cryo-EM reconstruction of the helical MeV nucleocapsid (PDB code 4UFT) [41]. Color coding as in (**A**), the RNA strand is shown in green; (**C**) Ribbon representation of two protomers of MeV Ncore (1–391). Color coding as in (**A**), the adjacent Ni + 1 protomer is shown in grey with the CTD arm colored in black. Left: view from the center of the helical structure. Right: top-down view.

Morbillivirus N proteins have an approximate length of 525 amino acids, and are mostly well sequence-conserved within the genus. Specifically, sequence alignments among different morbilliviruses have highlighted the existence of a conserved and ordered N-terminal core domain (Ncore) (spanning residues 1–391) that is followed by a far less conserved and intrinsically disordered N-terminal tail domain (Ntail) domain (residues 392–525) [30,36] (Figure 2A). Trypsin digestion of recombinant nucleocapsids has demonstrated resistance of Ncores to proteolysis. However, Ntails were sensitive to trypsin proteolysis, resulting in the conversion of nucleocapsids into rigid herringbone-like structures with a diameter of approximately 20 nm and a shortened pitch of 5 nm, compared to approximately 6.4 nm measured for native nucleocapsids that were found in infected cells [37,38,39]. Native MeV genomes present in viral particles have been reconstructed by cryo-electron microscopy, and show a flexible left handed herringbone structure similar to that described previously for recombinant nucleocapsid. However, structures of a larger diameter (30 nm) were also found that were coated by an additional layer of the viral M protein [40]. The physiological significance and/or functional importance of these matrix-wrapped structures is untested at present.

Providing a new level of insight into the molecular organization of the viral genome, a recent cryo-EM study has solved the MeV nucleocapsid structure in near-atomic resolution after removal of the Ntail domains (4.3 Å) [41] (Figure 2B). The Ncore domain of each N protein protomer consists of two globular domains, NTD (37–265) and CTD (266–372). Those domains are linked together by a short hinge region, and are flanked by N- and C-terminal extensions, the NT-arm (1–36) and the CT-arm (373–391). The high resolution reconstruction revealed that N protein oligomerization is mediated by the exchange of the NT-arm of a given Ni protomer with the CTD of the subsequent protomer (Ni + 1), while the CT-arm of the Ni protomer rests on top of the preceding Ni − 1, positioning the bulk of Ntail outside of the nucleocapsid by threading it between adjacent turns of the helix (Figure 2C). The RNA was found to wind around the nucleoprotein helix, populating the cleft between the NTD and CTD.

**Figure 3:**
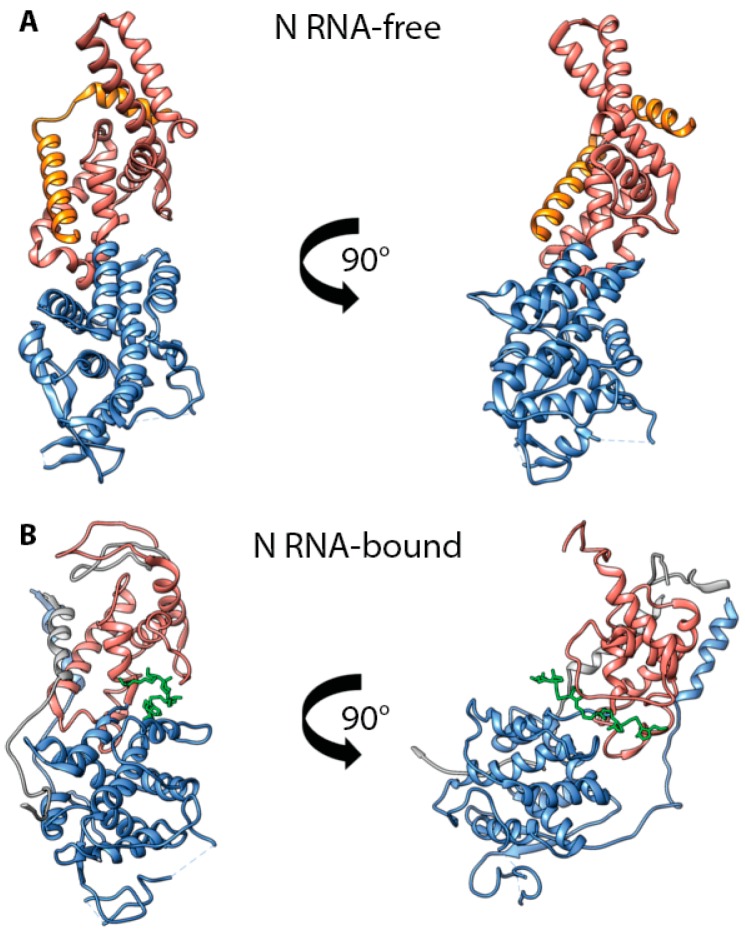
MeV N0-P interactions. (**A**) Ribbon representation of the crystal structure of a monomer of MeV N (residues 21–425) in complex with P (residues 1–48) (PDB code 5E4V) [42]. (**B**) Ribbon representation of the crystal structure of an MeV nucleocapsid protomer [41]. Both structures are oriented using the first CTD alpha helix as a reference. Color coding: NTD in blue, CTD in salmon, P residues 1–48 in orange, RNA is shown in green. Adjacent interacting NT- and CT-arms are rendered in grey.

Whereas the N protein has a strong tendency to oligomerize and bind RNA, it is imperative that a sufficiently large pool of monomeric, RNA-free N (N0) proteins can be accumulated in infected cells before the viral RdRp commits to replicase mode, to ensure uninterrupted cotranscriptional encapsidation of viral RNA. A recently solved X-ray structure of a chimeric protein of the MeV Ncore fragment (21–408) fused to the N-terminal fragment of the P protein (1–48) provided first insight into the organization of N0, facilitating a direct comparison of an RNA-bound and RNA-free morbillivirus N protein [42]. This structure revealed a direct interaction of P protein residues 1–48 with Ncore, specifically with the first alpha helix present in the N-CTD (Figure 3). Moreover, structure comparison with an N protomer embedded in assembled nucleocapsids shows that the position of P protein residues 1–48 in P-N0 complexes overlaps with that of the N-i NT-arm and Ni + 1 CT-arm in the helical assembly, conceivably sterically impeding the polymerization of P bound N proteins.

**Figure 4:**
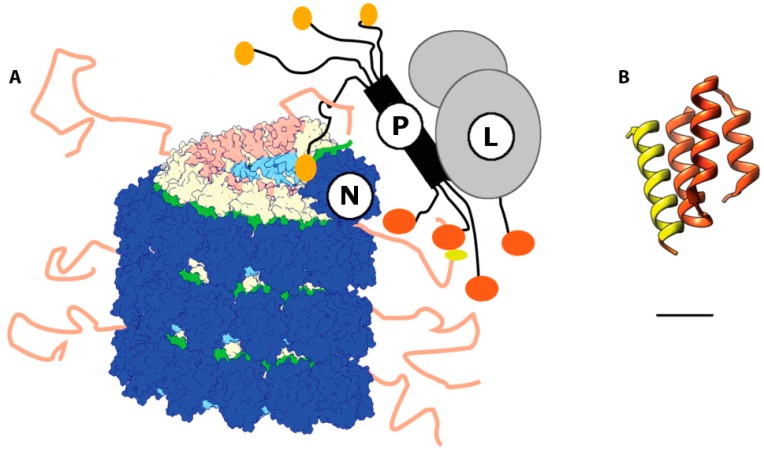
Model of the interaction between L, N, and P in the paramyxovirus RNA dependent RNA polymerase RdRp complex. (**A**) P binds L via its C-terminal domain. The P-L complex is recruited to the nucleocapsid through the interaction of the N-terminal domain of P (black and sand) with the N-CTD in the Ncore. Transient docking of P-XD (orange) and the MoRE in the Ntail (salmon) reduces the frequency of premature separation of the advancing polymerase complex from the RNP template. The P interaction with Ncore, possibly involving docking of the soyouz1 motif (sand) of P to N-CTD, locally opens the nucleocapsid and exposes the encapsidated RNA at the leading edge of the RdRp complex; (**B**) Ribbon representation of the crystal structure of a chimeric protein comprising PXD (dark orange, residues 457–507) and N (residues 477–505) (PDB code 1T6O) [48]. The α-MoRE of N has an alpha helical conformation spanning residues 487–503 and interacts with the second and third helixes of P-XD.

Although the most heterogeneous across different pathogens of the morbillivirus genus, the Ntail region contains three clusters of conserved residues, designated boxes 1 (400–420), 2 (489–506), and 3 (517–525) [30]. Overall, Ntail is mainly structurally disordered and residues (450–525) are thought to extend outward from the assembled nucleocapsid [43]. Its role in viral replication is not well understood; it was first proposed that Ntail is necessary for the recruitment of the RdRp complex to the nucleocapsid. Indeed, it has been shown in early studies of Sendai virus (SeV) that deletion of a fragment of Ntail leads to a significant loss of RdRp activity [44], and a C-terminal truncation of residues 495–525 of the MeV Ntail, which contain boxes 2 and 3, was found in subsequent work to cause a dramatic loss in RdRp activity in MeV minireplicon assays [45]. This observation appeared consistent with a role of Ntail in RdRp recruitment to the nucleocapsid, since box 2 contains a binding site for the P protein, the Molecular Recognition Element (MoRE), which specifically interacts with an X domain in the P protein (P-XD) that is located close to the C-terminus of the protein. MoRE assumes a molten globule conformation that transiently folds into short alpha helical structures, which are thought to establish the initial contact with P-XD. Subsequently, full conversion into a helical conformation is proposed to stabilize the interaction [46,47]. An X-ray structure of a recombinant MeV P-XD fused to a box 2-derived peptide provides high-resolution insight into this interaction [48] (Figure 4). Rather strikingly, however, further truncation of Ntail upstream of box 2 up to an additional 40 residues progressively restored RdRp activity in the MeV minireplicon system [49]. Expanding the reporter construct to full genome length in this study reduced transcription success, resulting in the current view that the MoRE P-XD interaction is, in fact, dispensable for RdRp recruitment to the genome. Rather, loading of the MeV RdRp complex onto the RNP template must be mediated by a direct interaction between P-L and Ncore in assembled nucleocapsids. Likely, truncating only the C-terminal region of the Ntails as done in the earlier studies masks access to Ncore, since the unstructured central Ntail regions are still present but cannot be ordered by the RdRp complex through interaction of P-XD with MoRE [49]. Once the polymerase is loaded on the template, MoRE P-XD binding is not mechanistically required for polymerization but appears to contribute to preventing premature separation of the advancing polymerase from the template through cycles of temporary binding and release (Figure 4A,B).

Two major phosphorylation sites have been identified near the C-terminal end of Ntail, flanking box 2: S479 and S510. Alanine substitutions of these serine residues reduced the transcriptional activity of MeV RdRp. However, the exact impact of phosphorylation on the interaction of box 2 with P-XD remains unknown [50]. Box 2 was also proposed as a binding site for a cellular chaperone, Hsp70 [51], and its cofactor Hsp40 [52], which could represent a secondary mechanism for activity modulation of the RdRp complex.

A comparable function was attributed to box 3, since it was suggested that this microdomain may stabilize the interaction between MoRE and P-XD and/or Hsp70 [45]. However, more recent work has revealed specific binding of the MeV M protein to box 3, spotlighting a central role in particle assembly by establishing a physical connection between the viral envelope and RNP genome [53,54].

### 2.2. P Protein

Morbillivirus P proteins show high sequence variability and feature a modular organization consisting of ordered and disordered domains [36]. While the P protein lacks enzymatic activity, it has a central role in replication as an essential polymerase co-factor, mediating loading of the complex onto the template and contributing to prevent premature termination of the advancing polymerase.

**Figure 5:**
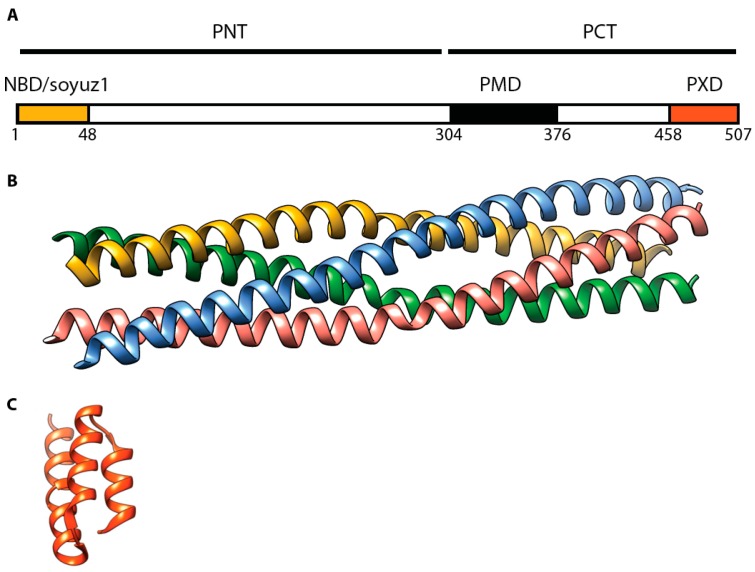
MeV P protein architecture. (**A**) Domain organization of the P protein. PMD: multimerization domain (residues 304–376) [56]; NBD: N0 binding domain (residues 1–48), including a soyuz1 motif [42,65]; PXD: N-tail binding domain (residues 459–507) [66]; (**B**) Ribbon representation of a PMD crystal structure (PDB code 3ZDO) [56]; (**C**) Ribbon representation of P-XD crystal structure (PDB code 1 OKS) [66].

Sequence alignments of morbillivirus P proteins suggested the presence of a coiled-coil oligomerization domain spanning residues 304–369 [55]. This notion was confirmed through crystallization of this region derived from the MeV P protein [56] (Figure 5). The recent structural work on MeV P corroborated earlier work studying RPV P, since sequential deletions of this region spotlighted RPV P residues 316–347 as required for oligomerization in a yeast two-hybrid assay. Furthermore, an RPV P fragment spanning residues 316 to 382 spontaneously tetramerized in solution and was predicted to assemble into a coiled-coil structure [57,58].

The RPV P protein was shown to interact specifically with the leader sequence of the viral genome, contributing to correct polymerase positioning for the initiation of polymerization. The oligomerization domain (316–382) was suggested to assume a pivotal role in this interaction, whereas phosphorylation of S88 prevented binding [59]. The phosphorylation state of RPV P may also modulate transcriptase versus replicase activity [60]. While an equivalent regulatory mechanism has not been demonstrated for MeV P yet, it is noteworthy that phosphorylation of MeV P residues S86 and/or S151 downregulates viral transcriptional activity [61].

In addition to facilitating L protein interaction with the genome, the P protein has co-chaperone function required for proper L protein folding, since transient expression of L in the absence of P greatly reduces L steady state levels [62]. Likely reflecting a mechanistic theme common to RNA virus polymerases [63], it was recently suggested that also the MeV P protein may be involved in recruiting cellular Hsp90 to the L protein [64].

The L binding domain of MeV P was mapped to the C-terminal PCT domain (residues 231–507) in yeast two hybrid studies [67]. However, comparison of candidate L binding domains of MeV and SeV P suggests that the actual interaction site is most likely located downstream of P residue L342 [56]. Although the precise molecular nature of the binding domain to MeV L remains unknown, it is most likely located immediately downstream of the oligomerization domain. This hypothesis is supported by three lines of evidence: (i) yeast two hybrid studies with RPV RdRp components have demonstrated that the interaction of RPV L with P involves P residues 347 to 490 [68]; (ii) this RPV P candidate binding region is able to engage in heterologous complexes between RPV and PPRV [33]; and (iii) outside of the morbillivirus genus, the P interaction domain with L was mapped downstream of the oligomerization domain for polymerase complexes of the human parainfluenza viruses type 2 and 3 and SeV [69,70,71].

### 2.3. L Protein

The large protein was originally named based on its high molecular weight of approximately 250 kDa. The protein harbors all enzymatic activities necessary for RNA transcription and replication: Phosphodiester bond formation, mRNA capping and methylation, and mRNA polyadenylation [72,73,74,75,76].

Paramyxovirus L proteins of morbilliviruses share a similar overall organization and specific functional motifs in particular are highly conserved [77]. Specifically, sequence alignments of different morbillivirus L proteins have suggested that two regions of high variability, residues 607–650 and 1695–1717, respectively, act as linker domains that separate three large regions (LR I to LR III) [78,79]. L proteins of RPV and MeV harboring large polypeptide insertions in the downstream variable region maintained substantial RdRp activity, while insertions into the 607–650 junction abolished bioactivity [79,80]. These results suggested that the L protein is conceived of at least two folding domains. It remained unclear, however, whether these domains are truly independently folding-competent or assume a native conformation only when synthesized as a single polypeptide. Information suggesting that synthesis as a single polypeptide is mandatory came from related vesicular stomatitis virus (VSV) L: like MeV L, VSV L also tolerates large polypeptide insertions into an interdomain region [81], but these VSV L subunits were unable to reconstitute a functional polymerase when expressed individually [82]. Addressing this question for paramyxovirus L, independently expressed domains LR I/II and LR III were found capable to restore activity through transcomplementation, but only after protein dimerization tags were added to the L fragments [29]. Successful transcomplementation could also be demonstrated for Nipah virus and RSV derived L fragments in that study, but heterotypic fragments failed to complement. These data demonstrated that paramyxovirus L proteins are composed of truly independent folding subunits that share a specific, but low-affinity domain interface.

**Figure 6:**
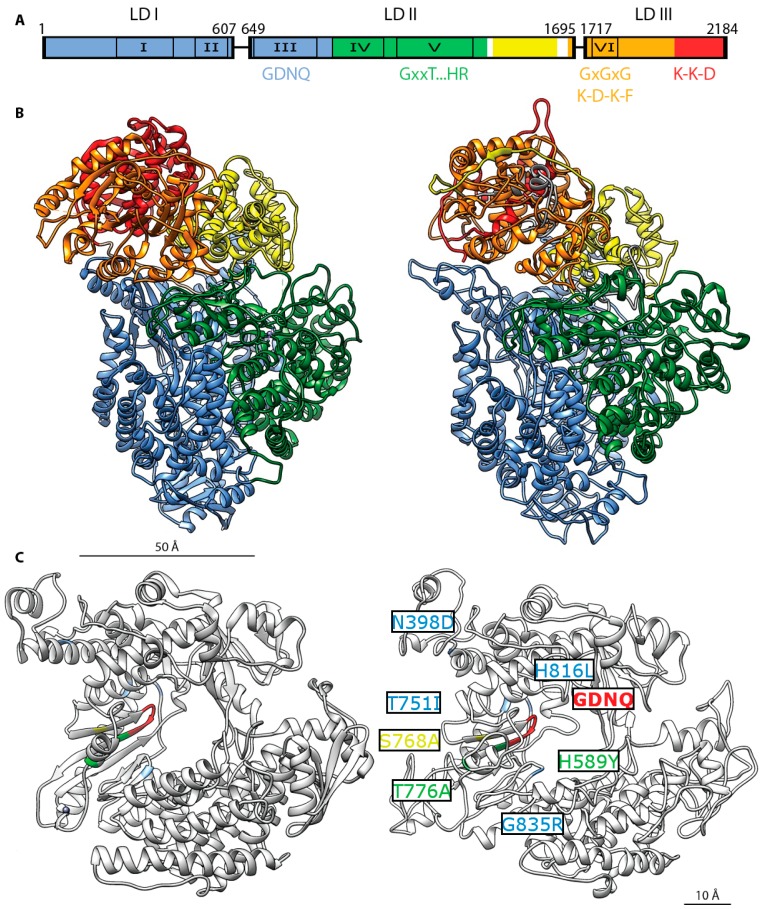
MeV L protein architecture. (**A**) Organization of three conserved domains (LD I–III) separated by two variable regions in the morbillivirus L protein (linker I: residues 607–649, linker II: residues 1695–1717) [29,78]. Six, conserved regions (CRs) present in all mononegavirales L proteins are numbered I to VI: residues 217–408, residues 495–599, residues 653–876, residues 927–1092, residues 1129–1376, and residues 1754–1831 respectively [83]. Color coding of the functional domain organization based on VSV L [97]: RdRp domain in blue, residues 1–924, capping domain in green, residues 925–1416, connector domain in yellow, residues 1439–1634, methyltransferase domain in orange, residues 1680–2018 and C-terminal residues 2019–2184. Specific conserved motifs involved in enzymatic activity are highlighted: GDNQ (residues 772–775), RdRp activity [76,98]; GxxT … HR (residues G1214, T1217, H1288, R1289, putative polyribonucleotidyl-transferase activity [95,99]; GxGxG (residues G1788, G1790, G1792), putative S-adenosyl-l-methionine binding site [100]; K-D-K-E (residues K1766, D1881, K1917, E1954), putative methyltransferase activity [91,92]; K-K-G (residues K2169 K2173 G2176), putative guanylyltransferase activity [88]; (**B**) Left: Ribbon representation of the cryo-EM structure of the VSV L protein [97]. Functional domains are rendered with the same color pattern. Right: homology model of the MeV L protein (derived from the Edmonston strain) rendered using the Swiss model server on the basis of the density maps released for VSV L [101]; (**C**) Mapping of the resistance mutations of the allosteric morbillivirus polymerase blocker ERDRP-0519 class [18,102]. Close-up view of the VSV L (left) and MeV L (right) polymerase catalytic site. The GDNQ tetrad is rendered in red. VSV L residues corresponding to resistance hotspots in MeV or canine distemper virus (CDV) L are rendered in yellow (MeV residue S768A), blue (CDV residues T751I, H816L, and G835R) and green (MeV and CDV residues H589Y and T776A) [102]. Corresponding residues on VSV L are rendered using the same color pattern.

Alignments of the L proteins from different members of the mononegavirales furthermore highlighted six conserved regions (CR I–VI) in the linear sequence of L [83,84] (Figure 6). Based on sequence data and structural analyses, most of the conserved regions were assigned a functional role in the RdRp complex. CR I (MeV 217–408) has been shown to be involved in the interaction with MeV and RPV P [62,64,68,85], and it was also proposed to mediate a putative homo-oligomerization [85]. CR II (MeV 495–599) and III (MeV 653–876) contain subdomains shared by all L polymerases [83]. In particular, a GDN tripeptide motif located in CR III is extremely conserved and is considered part of the catalytic center for phosphodiester bond formation [68,86]. CR V (MeV 1129–1376) and VI (MeV 1754–1831) are required for the cap synthesis and methylation of viral mRNAs. All paramyxoviruses replicate in the cytosol, which necessitates mRNA capping and cap methylation activities of the polymerase complex to ensure the synthesis of functional mRNAs and protect them from innate immunity. In mammalian cells, this post-translational step relies on distinct and sequential enzymatic activities: (i) RNA TriPhosphatase (RTPase) activity removes the 5′ phosphate of pre-mRNA; (ii) Guanylyl Transferase (GTase) activity uses a guanylyl triphosphate to transfer a guanylyl monophosphate on pre-mRNA (iii) Guanine-N7-MethylTransferase (GN7-MTase) followed by 2′O MethylTransferase (2′O MTase) fully methylates the new cap. RPV L has been shown to carry a GTase activity [87], that might involve a conserved C-terminal K-K-G motif [88], and an RTPase activity has been recently described [89,90]. Fragment (717–2183) has shown detectable GN7-MTase activity [91] and multiple sequence alignments also revealed the presence of K-D-K-E and GxGxG motifs described as involved in 2′O MTase activity [83,92,93]. Furthermore, a domain near the C-terminus of the L protein of human parainfluenzavirus type 2 shares homology with cellular GTases and was shown to be required for viral mRNA synthesis [94]. Nevertheless, the necessity for the L protein of morbilliviruses to carry its own enzymatic activities opens the alternative possibility that viral cap synthesis and methylation may diverge from the cellular pathway, since morbillivirus L also contain residues characteristic of an unconventional polyribonucleotidyl-transferase (PRNTase)-driven capping mechanism found in rhabdoviruses [95]. Further investigation is required to fully appreciate the morbillivirus capping mechanism.

Currently, no high-resolution structural data are available for full-length L proteins of any member of the paramyxoviridae. However, the crystal structure of a C-terminal fragment of the L protein of human metapneumovirus (HMPV) was recently solved, which encompasses CR VI [88] along with VSV CR I [96]. Furthermore, a cryo-electron microscopy reconstruction revealed the structure of VSV L in near atomic resolution [97] (Figure 6). This reconstruction revealed that the polymerase domain of VSV L comprises CRs I–III, whereas CRs IV–V form a capping domain and CR VI and the C-terminal residues build the MTase domain. These structures establish an important foundation for further structure-function analyses of the morbillivirus RdRp.

### 2.4. Mechanistic Implications

The high-resolution structures of the MeV N protein either complexed with RNA or in its free form has provided a first insight into a possible mechanism of how the RdRp complex may gain access to the genomic and antigenomic template RNA. Since the viral genome is tightly encapsidated in the left handed helical nucleocapsid, local RNP uncoiling must precede polymerization.

Juxtaposing the two N protein structures revealed an approximate 40° rotation between the N protein NTD and CTD upon assembly of N protein monomers into RNPs (Figure 3). This conformational change in MeV N is consistent with the information available for RNA-bound and -free N proteins of HMPV, a member of the Pneumoviridae closely related to the Paramyxoviridae [103], other paramyxoviruses such as parainfluenza virus 5 and Nipah virus, and distantly related members of the mononegavirales such as VSV and Ebola virus [104,105,106,107]. Interestingly, the end of the first CTD alpha helix of MeV contains a candidate phosphorylation site (residue T279) that is reportedly involved in regulating transcriptional activity and assembly of the helical RNPs [108]. Conceivably, the phosphorylation status of N residue 279 could affect the conformation of the CTD and modulate nucleocapsid conformation and accessibility of the RNA to the RdRp.

While we stand very much at the beginning of understanding the mechanism of morbillivirus RNA de-encapsidation, a direct role of N-terminal residues of the P protein in uncoiling of the RNP has been recently shown for mumps virus (MuV), a member of the rubulavirus genus in the paramyxovirus family [109]. The uncoiling event has a positive impact on MuV RdRp activity, since this study demonstrated that the presence of these P-NTD fragments enhanced RdRp activity in MuV minireplicon assays, supporting the notion that P directly interacts with Ncore and that this Ncore/P-NTD interaction may drive local exposure of the encapsidated viral RNA ahead of the advancing RdRp complex.

Morbillivirus RNA replication and transcription is considered to start at the 3′ extremity of the viral genome. On left handed nucleocapsids, these 3′ ends are buried in the terminal nucleoprotein protomers, which lack contact of their CTDs with adjacent NT-arms. The absence of a preceding N protomer consequently leaves hydrophobic patches of the first two alpha helixes of the MeV N-CTD vacant, rendering them easily accessible to the P protein [41,42] (Figure 5B). The ensuing initial P-NTD/N-CTD interaction may then lead to the observed shift/rotation in the N conformation and the local release or exposure of RNA to the RdRp catalytic core. An additional line of evidence comes from the recent demonstration that the MeV RdRp complex maintains transcriptase and replicase activity in the absence of MoRE and P-XD, provided a sufficiently large section of the unstructured central Ntail region is also removed [49]. The direct interaction of P with Ncore for polymerization and P-mediated local uncoiling of the RNP template may therefore extend to MeV and possibly represent a conserved central mechanistic theme of the paramyxovirus polymerase machinery.

The high-resolution reconstruction of the assembled MeV has furthermore contributed to revealing the structural basis for the strict adherence of members of the morbillivirus genus to the “rule of six” of genome length. Morbillivirus genomic and antigenomic promoters have a bipartite organization, such that the actual promoter elements are formed by nucleotides juxtaposed next to each other only in adjacent turns of the RNP helices [110,111,112,113]. Encapsidation imposes on the RNA a regular pattern or phase of base orientation relative to the individual N protomers: of the six nucleotides covered by each MeV N protomer, only the first, fifth, and sixth base are orientated outwards, while the second to fourth base are in an inwards orientation and therefore not available for promoter function [23,41,114]. The formation of both functional genomic and antigenomic promoter elements therefore mandates that the correct phase of the viral RNA relative to the encapsidating N proteins is preserved, which can only be achieved when genome length variations equal a multiple of six. Adherence to this rule is critical when designing recombinant morbillivirus genomes, since only recombinants with rule of six-compatible genomes can be successfully recovered.

## 3. Measles Therapeutics

Modern antiviral drug development commences with the identification of clinically valuable druggable targets that can be meaningfully pursued in hit discovery campaigns. Mononegavirales RdRp complexes offer a particularly deep and fruitful target pool based on subcellular localization in infected cells, spatial organization, and enzymatic activities that lack a cellular equivalent. These factors reduce the potential for undesirable cytotoxic or off-target effects. In addition, overall RdRp structure and specific functional sequence motifs are well conserved across different clinically relevant members of the paramyxovirus family, suggesting that it may be possible to broaden the anti-paramyxovirus indication spectrum of a promising, newly developed drug scaffold through the generation of relaxed analog libraries or structure-informed adaptation of the scaffold against a different paramyxovirus RdRp target.

In addition, the formation of active RdRp complexes depends on the contribution of host cell cofactors, providing a second layer of potential antiviral targets in a host-directed antiviral program. Examples of cellular factors that were implicated in morbillivirus RdRp activity include actin [115] and cytosolic chaperones such as Hsp70 [52], and Hsp90 [63,64]. Although the promise of a broadened antiviral target range and low frequency of viral resistance are correctly recognized as major assets of host-directed antiviral approaches [116], unacceptable toxicity profiles stall in many cases clinical development for infectious disease indications. As a case in point, the fundamental role of the actin cytoskeleton and key chaperones in eukaryotic cell biology will likely prevent meaningful therapeutic targeting of these factors for an anti-paramyxovirus indication.

Effective vaccine prophylaxis is in place against two major pathogens of the morbillivirus family, MeV and CDV. A third member of the genus, RPV, was declared eradicated in 2011 [117,118,119] after a globally coordinated vaccination campaign, and measles is currently targeted for global eradication [120]. These achievements raise the question of whether anti-morbillivirus therapeutics could make a meaningful contribution to disease management and the overall goal of global measles elimination.

After a dramatic drop in worldwide measles deaths in the first years after the global MeV control campaign was initiated, mortality rates bottomed out at approximately 100,000 cases annually in recent years [121]. Contributing factors are the extremely high infectivity rate of the virus that necessitates two doses of the vaccine and a vaccination coverage of essentially 100% of the pediatric population to fully suppress sporadic outbreaks [10,122], the dependence on an uninterrupted cold chain and trained health care personnel on the ground to deliver the vaccine, and, increasingly, parental refusal of vaccination based on safety concerns and/or religious beliefs [12,13]. These obstacles test political will and public resolve, especially in a scenario of a prolonged endgame of global eradication. Since measles is typically among the first vaccine-preventable disease to re-emerge in a geographic area when coverage drops and anti-MeV immunity provides cross-protection against zoonotic CDV infection [123,124], it is furthermore not reasonably conceivable that measles vaccination could ever be discontinued, calling for a continued global commitment to perpetually maintain high vaccination coverage rates.

In this setting, antivirals may support eradication efforts by creating an anti-MeV platform in conjunction with vaccine prophylaxis [15,16]. However, MeV is predominantly immunopathogenic [125,126], thus shifting the therapeutic window towards the prolonged 10-day incubation period and prodromal phase preceding symptomatic disease. Effective post-exposure prophylaxis of family members and social contacts of confirmed measles index cases may reduce transmission in endemic areas [127] and prevent reintroduction of the virus into geographical areas such as the United States, in which endemic transmission has been interrupted even though herd immunity has fallen below sterilizing coverage [128].

### 3.1. RdRp Inhibitors

Post-exposure prophylactic use in a predominantly pediatric patient population defines a very restrictive drug profile of a clinical candidate. In our view, the successful therapeutic must be shelf-stable at ambient temperature, orally bioavailable, amenable to cost-effective production using existing manufacturing technologies, and display an excellent safety profile. These diverse features are best met by a small-molecule therapeutic [129]. The nucleoside-analog Ribavirin has been used experimentally for the treatment of persistent measles infection [130], but limited efficacy and high toxicity exclude its use for post-exposure prophylaxis [131,132].

Representing one of the most advanced small-molecule morbillivirus inhibitors available to date, we have recently identified and chemically developed [133,134,135] an allosteric RdRp inhibitor that targets the L protein of MeV and CDV and suppresses polymerase activity [18]. The lead compound, ERDRP-0519, is orally bioavailable, enjoys excellent pharmacokinetic and toxicity profiles in small animal models, and effectively suppresses lethal CDV disease in ferrets when dosed following a post-exposure prophylactic regimen [18]. Treated animals mounted a robust immune response against CDV and were fully protected against subsequent challenge with a lethal virus dose. When viral escape was induced experimentally, resistance hot-spots concentrated in L regions CR II—IV, predominantly clustering around the catalytic site of the L polymerase domain (Figure 5C) and in two cases immediately flanking the conserved GRD motif [102,116]. In the CDV ferret model, all escape mutants were either strongly attenuated or displayed drastically reduced transmission success [18], suggesting that clinical or veterinary use is unlikely to induce the rapid emergence of pre-existing resistance in circulating virus strains.

### 3.2. Inhibitors of Paramyxovirus L Capping Activity

Other substructures of the morbillivirus RdRp complex were less well harnessed for therapeutic use to date. Guided by the growing insight into the structural organization of the mononegavirales polymerase complex, however, several promising target candidates were entertained against a number of different paramyxovirus polymerases. For instance, a recently developed compound, AZ-27, targets the RSV L protein, prevents the initiation of RNA synthesis for both transcription and replication, and, based on resistance data, is thought to restrict conformational flexibility of the RSV RdRp complex by targeting a hinge region between the capping and methyltransferase domains [136,137].

The mRNA capping activity of RSV L is proposed to be blocked by compound D [138]. In the presence of this compound, nascent mRNA transcripts are not capped when their 5′ ends reach the RdRp capping domain, preventing further mRNA elongation and leading to the accumulation of short, uncapped viral mRNA fragments [139].

Conceivably, the methyltransferase activity located in the CR VI of the paramyxovirus L protein presents another promising target candidate. This region harbors conserved motifs of a putative S-adenosyl-l-methionine transferase domain [92], may be readily druggable, since MTase inhibitors such as S-adenosyl-l-homocysteine derivates selectively inhibit methyltransferase activity of dengue virus (*Flaviviridae* family) [140].

### 3.3. Therapeutic Targeting of the Paramyxovirus N and P Proteins

In addition to targeting the L protein directly, RdRp activity can also be effectively suppressed by compounds docking to the viral N and/or P proteins. Compound RSV-604, for instance, reportedly targets the N protein and reduces both RNA synthesis and the infectivity of progeny virions [141]. The structural characterization of RSV P protein nucleocapsid interface has unearthed another attractive druggable target, since a conserved C-terminal phenylalanine residue of the P protein was shown to transiently dock into a defined pocket formed by the N protein [142], thus stabilizing the interaction of the advancing polymerase complex with N:RNA template in a functional equivalent to the MoRE P-XD interaction of the morbilliviruses. Although small molecule-mediated targeting of protein-protein interfaces is often challenging [143], the unusually small protein-protein interface area, moderate binding affinity, and highly dynamic nature of the interaction render the RSV P interface with the nucleocapsid highly druggable. A pilot drug screen was attempted that applied an in silico campaign to the problem, but this effort did not yield convincing proof-of-concept, since hit candidates were barely active (50% inhibitory concentrations >120 μM) and cytotoxic and active concentrations remained within an unacceptable two-fold range of each other [142].

Homology models of the paramyxovirus L proteins built based on the near-atomic resolution structure of VSV L have advanced the overall structural understanding of the polymerase complex. However, the confidence of these models is still insufficient to mount meaningful virtual drug screening campaigns. To establish a valid foundation for structure-based drug discovery, the problem needs to be alleviated through the generation of true atomic resolution substructures, as exemplified for instance by the crystal structure of the HMPV MTase domain or the partial RSV N-P complex. Despite the current limitations, the structural information generated in the past two years alone has in combination with resistance mapping greatly furthered the mechanistic characterization of existing inhibitors such as the ERDRP-0519 MeV inhibitor class. While physical drug screens are therefore anticipated to remain imperative in the foreseeable future for the de novo identification of viable hits, we anticipate structure-informed lead development, mechanism of action characterization, and proactive design of drug variants with improved resistance profile to greatly profit from the mounting body of structural insight into the paramyxovirus RdRp complex.

## 4. Conclusions

Faced with a possible prolonged endgame of global measles eradication, novel therapeutics for effective post-exposure prophylaxis could substantially accelerate progress towards the overall goal of viral elimination. Propelled by a rapidly growing body of information concerning the functional and spatial organization of paramyxovirus polymerases in general and MeV RdRp in particular, the MeV polymerase complex emerges as a particularly promising target for novel therapeutics. Providing proof-of-concept for the validity of the approach, an orally efficacious clinical candidate with desirable drug profile was recently developed. To augment MeV eradication, it will likely be possible to also adapt a number of current antiviral strategies directed against the polymerases of related paramyxoviruses to the MeV problem. Pursuing the polymerase complex through structurally and mechanistically distinct drug candidates will reduce the risk of clinical failure of a single scaffold and open opportunities to further reduce the frequency of viral escape and capitalize on synergistic effects through combination therapies.
